# Lost in translation: interferon-stimulated genes targeting flavivirus protein synthesis

**DOI:** 10.1128/jvi.00303-26

**Published:** 2026-06-04

**Authors:** Marion Cannac, Jim Zoladek, Sébastien Nisole

**Affiliations:** 1 Institut de Recherche en Infectiologie de Montpellier (IRIM), University of Montpellier, CNRS, INSERM 131821, Montpellier, France; 2 Pathogenesis and Control of Chronic and Emerging Infections (PCCEI), INSERM, EFS, University of Montpellier 728323, Montpellier, France; 3 Centre Armand-Frappier Santé Biotechnologie, Institut National de la Recherche Scientifique (INRS) 14851 , Laval, Quebec, Canada; New York University Department of Microbiology, New York, New York, USA

**Keywords:** *Orthoflavivirus*, interferon-stimulated genes (ISGs), translation control, viral RNA, innate immunity, ribosome, RNA structure, restriction factors

## Abstract

Flaviviruses are positive-sense RNA viruses that rely entirely on host translation machinery to express their genome, making protein synthesis a central point of vulnerability. Interferon-stimulated genes (ISGs) exploit this dependence through diverse and mechanistically distinct strategies. PKR and IFIT proteins interfere primarily with translation initiation by targeting initiation factors or cap recognition, whereas SLFN11 and SAMD9L impair elongation through codon- and tRNA-dependent mechanisms. In parallel, ZAP, SHFL, and ISG20 inhibit translation by excluding viral RNAs from ribosomes or promoting their degradation. These antiviral activities highlight that ISG-mediated restriction is often highly selective, targeting viral RNAs on features such as cap structure, nucleotide composition, codon usage, and RNA folding. As a result, translation emerges as a central interface of host–virus conflict, where subtle differences between viral and cellular mRNAs can be exploited to achieve potent antiviral effects while preserving host protein synthesis. This minireview highlights recent advances in the identification and characterization of ISGs that restrict flavivirus protein synthesis and integrates them into a unified framework based on their primary mechanism of action. Emphasizing how host defenses target multiple stages of translation provides a conceptual basis for understanding how innate immunity controls flavivirus replication and highlights viral translation as a promising target for selective antiviral strategies.

## INTRODUCTION

**Fig 1:**
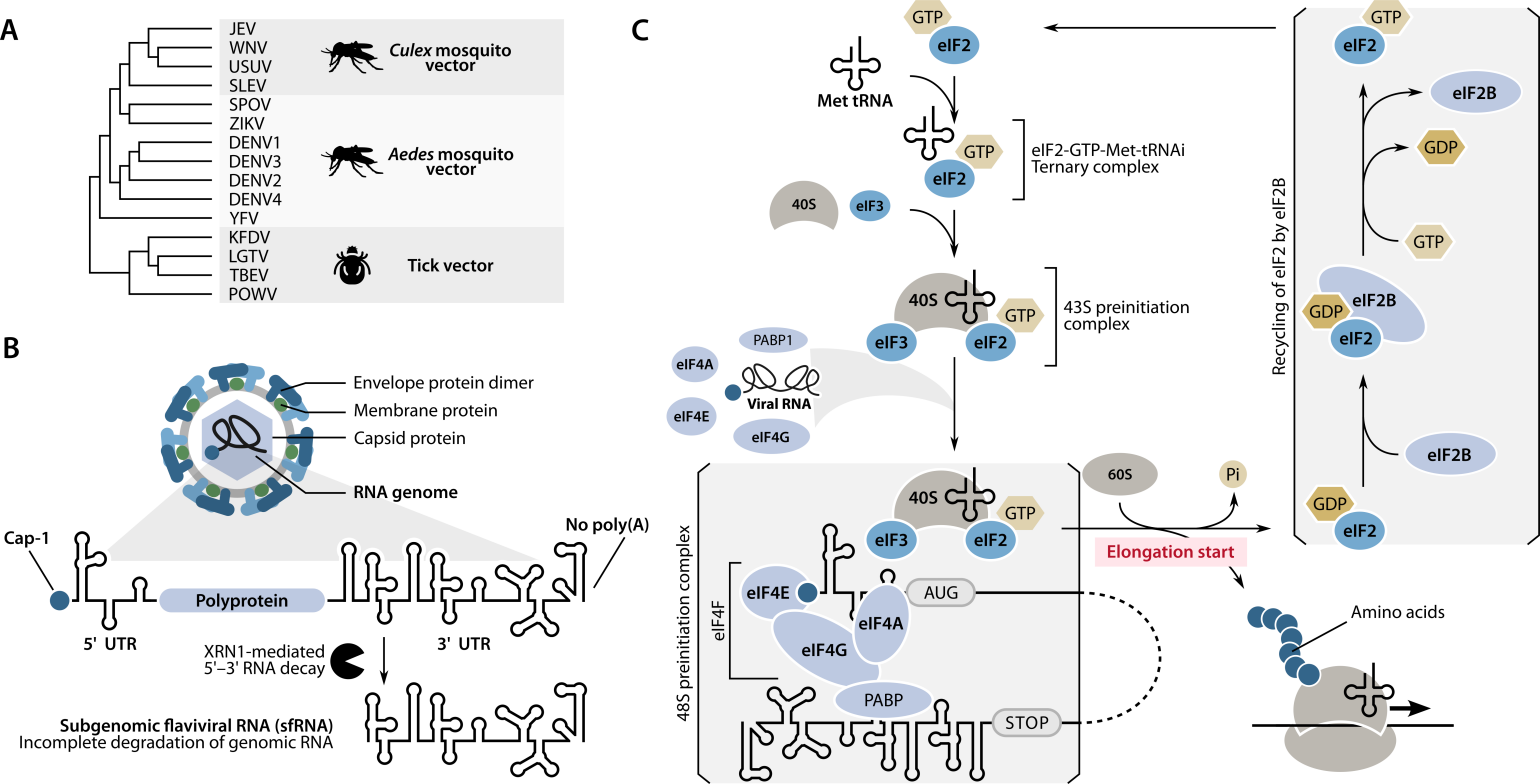
*Orthoflavivirus* phylogeny, genome organization, and translation initiation. (**A**) Schematic phylogeny of major mosquito- and tick-borne orthoflaviviruses pathogenic to humans. Principal arthropod vectors, including *Aedes* and *Culex* mosquitoes and ixodid ticks, are indicated. Branch lengths are not to scale. Viruses shown include Japanese encephalitis virus (JEV), Usutu virus (USUV), West Nile virus (WNV), St. Louis encephalitis virus (SLEV), Spondweni virus (SPOV), Zika virus (ZIKV), dengue virus serotypes 1-4 (DENV1–4), yellow fever virus (YFV), Kyasanur forest disease virus (KFDV), Langat virus (LGTV), tick-borne encephalitis virus (TBEV), and Powassan virus (POWV). (**B**) Schematic representation of the *Orthoflavivirus* virion and genome organization. The enveloped particle consists of a host-derived lipid bilayer containing the envelope (E) and membrane (M) proteins, and a nucleocapsid composed of capsid (C) protein and a single-stranded positive-sense RNA genome. The genome carries a 5′ cap-1, lacks a 3′ poly(A) tail, and encodes a single open reading frame that is translated into a polyprotein. Conserved RNA elements within the 5′ and 3′ UTRs are indicated. Subgenomic flaviviral RNAs (sfRNAs) are generated by incomplete degradation of the genomic RNA by the host 5′–3′ exonuclease XRN1. Structured RNA elements in the 3′ UTR stall XRN1, resulting in accumulation of sfRNAs that contribute to viral replication and modulation of host responses. (**C**) Model of *Orthoflavivirus* translation initiation. The eIF4F complex binds the 5′ cap and cooperates with PABP associated with the 3′ UTR to promote mRNA circularization. eIF2 is recycled by eIF2B through GDP–GTP exchange, enabling formation of the ternary complex (eIF2-GTP-Met-tRNAi). This ternary complex associates with the 40S ribosomal subunit and eIF3 to form the 43S preinitiation complex. The 43S complex is recruited to the mRNA via eIF4F, forming the 48S preinitiation complex, which scans the 5′ UTR to locate the start codon. Upon AUG recognition, GTP hydrolysis triggers the release of initiation factors, joining of the 60S subunit, and formation of the 80S ribosome, marking the onset of elongation.

Flaviviruses are positive-sense RNA viruses belonging to the genus *Orthoflavivirus* within the family *Flaviviridae*. Many medically relevant members of this genus are arthropod-borne and transmitted to vertebrate hosts by mosquitoes or ticks, a feature that facilitates their maintenance in nature and broad geographic distribution. As a result, flaviviruses include some of the most widespread and clinically significant viral pathogens affecting humans (1). Over the past decades, the geographic range of several flaviviruses has expanded, driven by climate change, environmental change, and increased human mobility. Dengue virus (DENV), yellow fever virus (YFV), Zika virus (ZIKV), West Nile virus (WNV), and tick-borne encephalitis virus (TBEV) (Fig. 1A) together account for millions of infections annually, with clinical manifestations ranging from mild febrile illness to severe hemorrhagic or neurological disease (1). In parallel, other flaviviruses such as Usutu virus (USUV), Spondweni virus (SPONV), and Tembusu virus (TMUV) (Fig. 1A) have emerged or expanded in new regions, reflecting the evolving epidemiology of this genus (2–4). Despite their genetic and ecological diversity, flaviviruses share a conserved genome organization and replication strategy. Their 10–11 kb genome consists of a single-stranded, positive-sense RNA bearing a 5′ cap and flanked by highly structured untranslated regions (UTRs) (Fig. 1B). Both the 5′ and 3′ UTRs are essential for translation, replication, and RNA stability (5). In addition, partial degradation of the viral genome by the host exonuclease XRN1, which stalls at specific structured elements within the 3′ UTR, generates small non-coding RNAs called subgenomic flavivirus RNAs (sfRNAs) (6) (Fig. 1B). These sfRNAs accumulate in infected cells and play key roles in viral pathogenesis and the modulation of host antiviral responses (7). The flaviviral genome encodes a single polyprotein, which is processed into three structural and seven non-structural proteins. Upon release into the cytoplasm, the viral RNA serves directly as mRNA to initiate translation and the infectious cycle. Replication occurs in membrane-associated complexes derived from the endoplasmic reticulum, with newly synthesized RNA used for further translation, replication, or packaging (1, 8).

The first line of defense against flavivirus infection is the innate immune response, particularly the type I interferon (IFN) system (6). IFN signaling induces hundreds of interferon-stimulated genes (ISGs), whose products collectively restrict viral replication (9, 10). Among the stages of the viral life cycle targeted by ISGs, protein synthesis is especially vulnerable, since flaviviruses, as obligate intracellular parasites, rely entirely on host ribosomes, translation factors, and tRNA pools to produce viral proteins. This absolute dependence makes translation highly sensitive to perturbation, with even modest disruptions immediately affecting viral replication.

In eukaryotic cells, most mRNAs are translated through a cap-dependent initiation mechanism that begins with recognition of the 5′ cap by the eukaryotic initiation factor 4F (eIF4F) complex (11). This heterotrimeric complex, composed of the cap-binding protein eIF4E, the RNA helicase eIF4A, and the scaffold protein eIF4G, recruits the 43S pre-initiation complex through interactions with eIF3 and promotes mRNA circularization via binding to poly(A)-binding protein (PABP) (11, 12). eIF4A further facilitates ribosomal scanning by unwinding secondary structures in the 5′ UTR. In higher eukaryotes, the 5′ cap typically corresponds to a cap-1 structure (m7GpppNm), in which the first transcribed nucleotide is 2′-O-methylated, a modification that enhances translation efficiency and contributes to self/non-self-discrimination (13). A critical step in translation initiation is the formation of the ternary complex (TC), composed of eIF2 bound to GTP and initiator methionyl tRNA (Met-tRNAi), which is essential for assembly of the 43S pre-initiation complex and start codon recognition. Because translation initiation relies on a limited set of host factors, it is highly sensitive to stress and antiviral signaling (14, 15).

Flaviviruses fully exploit this canonical translational machinery (Fig. 1C). Their genome carries a cap-1 structure generated by the viral NS5 protein, whose sequential N7 and 2′-O methyltransferase activities produce a 5′ cap that closely mimics host mRNAs (16–18). This viral capping is essential because, as viral replication occurs entirely in the cytoplasm, the RNA is not accessible to host nuclear methyltransferases. The resulting cap ensures productive engagement of the eIF4F complex and facilitates assembly of the translation initiation machinery. Structured UTRs and long-range RNA interactions further promote translation (5, 19), and, despite lacking a poly(A) tail, they recruit PABP through interactions with the 3′ UTR (20, 21) (Fig. 1C). At the same time, the strict dependence of flaviviruses on host ribosomes, initiation factors, and tRNA pools creates vulnerabilities that ISGs can target. Many ISGs restrict flavivirus replication by interfering with translation initiation, ribosome progression, RNA stability, or ribosome engagement, converging on translation as a central control point.

In this review, we focus on ISGs that inhibit flavivirus replication at the level of translation. We classify these ISGs based on their primary mechanisms: blocking initiation, hindering elongation or ribosome progression, excluding viral RNAs from ribosomes, or indirectly suppressing translation by reducing viral RNA abundance. By focusing on translational control, we aim to delineate how host defenses exploit this essential step in the flavivirus life cycle.

## INTERFERON-STIMULATED GENES THAT INHIBIT TRANSLATION INITIATION

Translation initiation is a particularly vulnerable step in the flavivirus life cycle. Because the incoming viral genome serves directly as a capped messenger RNA, any perturbation affecting ribosome recruitment or initiation complex formation has immediate consequences for viral protein production and, consequently, viral replication. Unlike later stages of translation, initiation is highly regulated and depends on a limited number of host factors, making it an efficient point of control for antiviral restriction. Several ISGs exploit this vulnerability by interfering with translation initiation through distinct but often complementary mechanisms. These range from global inhibition of initiation factor activity to selective exclusion of viral RNAs from the translational machinery.

### PKR (EIF2AK2)

Protein kinase R (PKR, also known as EIF2AK2, eukaryotic translation initiation factor 2 alpha kinase 2) is one of the most extensively studied ISGs with broad antiviral activity. PKR expression is strongly upregulated by type I IFNs, and its activation is triggered by binding to double-stranded RNA (dsRNA), a replication intermediate commonly generated during viral infection (22, 23).

**Fig 2:**
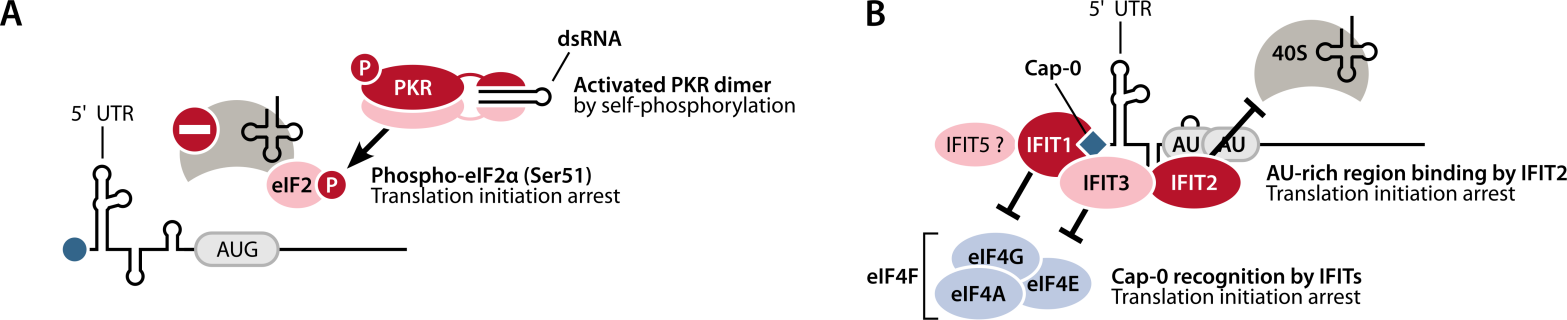
ISGs restricting *Orthoflavivirus* translation initiation. (**A**) Viral double-stranded RNA activates PKR, inducing its dimerization and autophosphorylation, which, in turn, phosphorylates eIF2α and suppresses translation initiation. (**B**) IFIT proteins bind non-2′-O-methylated (cap-0) viral RNAs and AU-rich or atypical sequence elements, preventing eIF4F recruitment and 40S ribosome loading, thereby inhibiting cap-dependent translation. Flaviviruses encoding a 2′-O-methylated cap-1 via NS5 partially evade this restriction.

Binding of dsRNA induces PKR dimerization and autophosphorylation, enabling PKR to phosphorylate the α subunit of eukaryotic translation initiation factor 2 (eIF2α) at serine 51 (Fig. 2A). Under normal conditions, eIF2-GTP is hydrolyzed to eIF2-GDP after start codon selection and must be recycled by eIF2B to sustain translation initiation (Fig. 1C). Phosphorylation of eIF2α converts eIF2-GDP into a high-affinity inhibitor of eIF2B, preventing ternary complex regeneration and leading to global inhibition of translation initiation (24, 25) (Fig. 2A). This translational arrest is often accompanied by the assembly of stress granules (SGs), which sequester mRNAs and translation factors, further limiting protein synthesis (26).

Through these mechanisms, PKR exerts broad-spectrum antiviral activity against multiple RNA and DNA viruses, although many have evolved countermeasures to evade PKR-mediated restriction (27). While PKR activation depends on dsRNA, its antiviral outcome is largely determined by the ability of viral RNAs to remain efficiently translated under conditions of eIF2α phosphorylation. Other kinases, including HRI and PERK, also phosphorylate eIF2α as part of the integrated stress response, and flaviviruses such as USUV, WNV, and DENV-2 can modulate these pathways to dampen eIF2α phosphorylation or stress granule assembly, further influencing translation independently of PKR (28–30). Thus, PKR activation alone does not reliably predict antiviral outcome, reflecting the context-dependent impact of PKR on viral replication.

PKR restricts JEV, although the viral NS2A protein counteracts this effect by preventing eIF2α phosphorylation, thereby sustaining viral protein synthesis (31). WNV is sensitive to PKR in human cells (32, 33) and in mice (34), yet not in rodent fibroblasts, where PKR is not activated (35), indicating that PKR responsiveness may vary between host species and cell types. In contrast, DENV-2 is largely PKR-insensitive in murine and human cells, but no direct viral countermeasure has been described (28, 32). Interestingly, PKR can also exert proviral effects in certain contexts. For DENV-4 and ZIKV, PKR activation can be co-opted to promote replication (36, 37). In ZIKV infection, sfRNAs activate PKR and induce transient eIF2α phosphorylation, which dampens IFN and ISG expression and thereby favors viral replication (36). Structural features within the ZIKV 5′ UTR allow viral RNAs to partially escape PKR-mediated translational inhibition (37). These observations underscore that PKR’s impact on flavivirus replication is virus-specific, with different viruses using diverse strategies to evade or manipulate it. Some suppress PKR signaling, while others tolerate or exploit eIF2α phosphorylation, often via intrinsic RNA features that enable ribosome recruitment under stress. Consistent with this idea, several flaviviruses have been reported to initiate translation through cap-independent mechanisms when cap-dependent initiation is compromised (20, 38–41), although their contribution to PKR evasion remains unclear.

Despite decades of study, key questions remain. Systematic comparisons across flaviviruses are lacking, limiting our ability to define determinants of sensitivity, resistance, and antiviral efficacy. Comparative analyses in standardized systems, integrating measurements of viral replication, eIF2α phosphorylation, and stress granule dynamics in the presence or absence of PKR and related kinases, will be essential to distinguish conserved from virus-specific strategies.

Overall, PKR occupies a central yet context-dependent position among translation-targeting ISGs, integrating control of protein synthesis and stress responses. Developing a comparative framework will be critical to fully understand how PKR shapes flavivirus replication and host–virus interactions.

### IFIT proteins

Interferon-induced proteins with tetratricopeptide repeats (IFITs) constitute a key family of antiviral effectors that target viral protein synthesis at the level of translation initiation. IFIT proteins lack any known enzymatic activity and inhibit translation by directly binding viral RNAs and preventing their recruitment to the ribosome. The human IFIT family includes IFIT1, IFIT2, IFIT3, and IFIT5, all of which localize to the cytoplasm and are expressed at low or undetectable levels under basal conditions (42). IFIT1, formerly known as ISG56, and IFIT2, formerly ISG54, were among the first ISGs identified, highlighting their central role in innate antiviral defense (43, 44).

IFIT1 is the best characterized member of this family. It preferentially binds viral RNAs carrying a 5′ cap lacking 2′-O methylation, known as cap-0 (45). By occupying the cap structure, IFIT1 prevents binding of eIF4E and thereby excludes the eIF4F complex from viral RNAs, resulting in inhibition of translation initiation (46–48). This selectivity allows IFIT1 to discriminate viral RNAs from host mRNAs, which generally carry a cap-1 structure. Interestingly, IFIT3 enhances IFIT1-mediated antiviral activity by stabilizing IFIT1–RNA complexes, further strengthening translational inhibition (49) (Fig. 2B). In flaviviruses, the NS5 methyltransferase converts cap-0 into cap-1 by adding a 2′-O methylation to the first nucleotide, thereby protecting viral RNAs from IFIT1-mediated restriction. Experimental evidence demonstrates that IFIT1 strongly restricts flaviviruses lacking 2′-O cap methylation. This was first shown with WNV, where the NS5 E218A mutant is highly sensitive to IFIT1, whereas wild-type cap-1 viruses are largely resistant (45, 50). Similar observations have been reported for JEV and DENV, in which mutations in NS5 that abolish 2′-O methyltransferase activity lead to viral attenuation and enhanced sensitivity to type I IFN and IFIT1 (47, 51, 52).

In addition to recognizing cap-0 RNAs, IFIT1 has been reported to associate with the eIF3 translation initiation complex, particularly through the eIF3e subunit, resulting in a global translation inhibition in human cells (53). Although IFIT1 can affect eIF3 function, the contribution of this interaction to global versus virus-selective translation inhibition likely depends on cellular context and IFIT expression levels. This mechanism also underlies the repression of internal ribosome entry site (IRES)-mediated translation of hepatitis C virus RNA, while cap-dependent translation of host mRNAs remains largely unaffected (54). Given that flaviviruses can also engage cap-independent translation (20, 38–41), IFIT1-eIF3 interactions may similarly impair this process, particularly when cap-dependent translation is already compromised. However, despite this potential for broader translational control, available evidence supports a model in which IFIT1 restricts flaviviruses mainly through selective viral RNA targeting via cap recognition, rather than broad translation inhibition.

Other IFIT family members also contribute to antiviral defense, often through complementary or distinct mechanisms. IFIT2 is an RNA-binding protein with a preference for AU-rich sequences, a property required for antiviral activity against several RNA viruses (55) (Fig. 2B). In the context of flavivirus infection, murine IFIT2 contributes to the IFN-mediated restriction of WNV and protects mice from lethal challenge, although the exact mechanism underlying its antiviral effect remains unclear (56). IFIT3 displays both cofactor and independent antiviral roles. It enhances IFIT1-mediated translational inhibition (49) and independently restricts DENV-2 and ZIKV replication, limiting virus-induced cytopathicity in human cells (57, 58). IFIT5 has also been reported to possess antiviral activity similar to murine IFIT1, although its mechanism remains less well characterized (45) (Fig. 2B).

Collectively, the IFIT family exemplifies a host strategy that exploits subtle structural features of viral RNAs to selectively inhibit viral protein synthesis while sparing host translation. Their activity underscores translation as a critical vulnerability in the flavivirus life cycle and a central target of the IFN response. Beyond antiviral restriction, IFIT proteins have also been reported to modulate inflammatory pathways, reflecting their role as pleiotropic effector ISGs (42).

Although IFIT proteins clearly exploit RNA cap structure and sequence features to restrict viral translation, the rules governing virus-specific sensitivity, the functional interplay among family members, and the role of cofactors remain poorly defined. Addressing these points could illuminate how IFITs achieve selective antiviral effects and inform strategies to enhance their activity against flaviviruses.

## INTERFERON-STIMULATED GENES THAT INHIBIT TRANSLATION THROUGH RNA RECOGNITION AND RIBOSOME EXCLUSION

Beyond mechanisms that inhibit translation initiation, some ISGs restrict viral translation by directly recognizing viral RNAs and preventing their engagement with ribosomes. In these cases, inhibition of protein synthesis occurs upstream of RNA degradation and reflects an early exclusion of viral transcripts from the translational pool. This strategy allows rapid suppression of viral gene expression before extensive RNA decay takes place.

### ZAP (ZC3HAV1)

Zinc-finger antiviral protein (ZAP, also known as ZC3HAV1 or PARP13) is a well-characterized IFN-stimulated RNA-binding protein that restricts viral replication by selectively recognizing non-self features in viral RNAs and excluding them from the translational pool (59). ZAP is expressed as at least four isoforms, among which ZAP-S and ZAP-L are the best studied (60, 61). It was initially identified as a restriction factor for Moloney murine leukemia virus, where it markedly reduced cytoplasmic viral RNA without affecting nuclear RNA levels, pointing to a post-transcriptional mechanism (62). Subsequent studies established ZAP as a broadly acting antiviral factor targeting a wide range of RNA viruses (60, 61).

**Fig 3:**
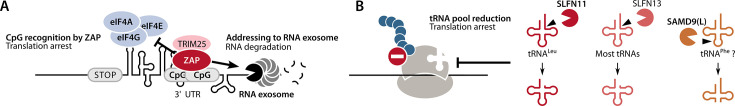
ISGs targeting translation elongation and ribosome progression. (**A**) ZAP binds CpG-enriched regions and other sequence or structural motifs within viral RNA, including elements in the 3′ UTR. Through interaction with TRIM25 and additional cofactors, ZAP inhibits the recruitment of host translation factors and recruits cellular RNA decay pathways, including the RNA exosome, leading to viral RNA degradation. (**B**) Schlafen-family ribonucleases (e.g., SLFN11 and SLFN13) and SAMD9/SAMD9L function as anticodon nucleases (ACNases) that inhibit translation elongation by cleaving specific tRNA subsets, thereby reducing the availability of functional tRNAs and suppressing viral protein synthesis.

ZAP recognizes viral RNAs primarily through its N-terminal CCCH-type zinc finger domains, which preferentially bind single-stranded RNAs enriched in CpG dinucleotides or other atypical sequence or structural features (63–66). These compositional biases are uncommon in host mRNAs and are therefore interpreted as signatures of non-self RNA. Upon binding its target RNA, ZAP assembles antiviral complexes that can inhibit translation, promote RNA decay, or both, depending on the viral context and available cofactors (62, 63, 67–69) (Fig. 3A).

Although ZAP is frequently associated with RNA degradation, translation inhibition is an early and primary outcome, preceding RNA decay (68). Mechanistically, ZAP can inhibit translation initiation by interacting with eIF4A and weakening the eIF4A–eIF4G interaction, thereby preventing efficient recruitment of ribosomes to target RNAs (68). These features allow ZAP to act selectively on viral RNAs while sparing host translation, highlighting RNA composition and structure as key determinants of sensitivity (Fig. 3A).

In flavivirus infection, ZAP displays virus-specific effects. ZAP binds the JEV genome through its zinc finger domains, with a strong interaction mapped to the 3′ UTR, leading to reduced ribosome loading and decreased viral translation (63). Consistent with its CpG-targeting activity, ZAP has little effect on DENV, which exhibits lower CpG dinucleotide frequencies (63), but efficiently restricts WNV and USUV, underscoring virus-specific determinants (70). The activity of ZAP against ZIKV remains context-dependent. While ZAP overexpression did not impair ZIKV replication in IFN-competent human cells (63), ZAP knockout studies in IFN-deficient Vero cells revealed a clear antiviral effect (71, 72). Taken together, these observations suggest that CpG content and RNA structural features are major determinants of flavivirus sensitivity, predicting that increasing CpG frequency in otherwise resistant genomes would sensitize them to ZAP.

The ZAP function is strongly shaped by its interaction with cellular cofactors. TRIM25 enhances ZAP-mediated inhibition of JEV translation, despite not directly binding viral RNA in this context, suggesting a role in stabilizing or activating ZAP-containing antiviral complexes (73) (Fig. 3A). In addition to translation inhibition, ZAP can recruit RNA decay machineries acting in both 5′–3′ and 3′–5′ directions. The 5′–3′ pathway typically involves the decapping enzymes DCP1 and DCP2 together with the 5′–3′ exonuclease XRN1, whereas 3′–5′ decay is mediated by the RNA exosome complex (63, 67–69). Interestingly, the involvement of XRN1 in ZAP-mediated restriction of JEV was shown to be dispensable, whereas knockdown of the exosome component EXOSC5 impaired the antiviral effect (63) (Fig. 3A). Whether the same mechanism applies to other flaviviruses remains to be determined.

Overall, ZAP exemplifies a class of ISGs that restrict viral replication by direct RNA recognition followed by exclusion from ribosomes, rather than by inducing global translational shutdown. By excluding viral RNAs from the translational pool, ZAP imposes strong selective pressure on viral genome composition and highlights how innate immunity exploits subtle sequence features to discriminate viral RNAs from host mRNAs. The viral and host factors that govern ZAP sensitivity, and the balance between translation inhibition and RNA decay, remain to be fully defined. In particular, the potential interplay between ZAP and sfRNAs represents an intriguing avenue for future investigation. sfRNAs bind multiple RNA-binding proteins involved in RNA surveillance and can inhibit XRN1, potentially modulating the efficiency of ZAP-mediated RNA degradation (74, 75). Understanding how sfRNAs influence ZAP effector functions may help explain virus-specific differences in ZAP activity and uncover mechanisms by which flaviviruses evade intrinsic antiviral defenses.

## INTERFERON-STIMULATED GENES THAT RESTRICT TRANSLATION ELONGATION OR RIBOSOME PROGRESSION

Beyond initiation, translation elongation is also vulnerable. The flaviviral genome encodes a single, long open reading frame that must be translated with high ribosome processivity to ensure sufficient production of viral proteins. Even modest perturbations in elongation dynamics can therefore result in a disproportionate decrease in viral protein output, with immediate consequences for genome replication and virion assembly. Several ISGs exploit this dependence by targeting ribosome progression or the ribosome–tRNA interface, thereby selectively impairing viral translation without directly affecting RNA abundance.

### SLFN11

Schlafen 11 (SLFN11) is an IFN-induced restriction factor that inhibits the replication of multiple viruses by interfering with translation elongation in a codon-dependent manner. Unlike IFIT proteins, SLFN11 does not bind viral RNA directly, but cleaves specific type II tRNAs, with a preference for tRNA^Leu^, thereby reducing the pool of available tRNAs and selectively impairing the viral translation (76, 77). Its antiviral activity is particularly pronounced against RNAs that rely on suboptimal codon usage and non-abundant tRNAs (Fig. 3B).

The human SLFN family comprises six members that vary in length and sequence, but all contain the characteristic Schlafen core domain (78). This domain includes a highly conserved zinc finger motif and the Schlafen box, which harbors a conserved Glu-Glu-Asp (EED) triad responsible for ribonuclease activity (79). SLFN proteins perform diverse roles in cellular homeostasis and cancer and have been characterized as regulators of immunity and restriction factors of multiple viruses (78, 80). Although the active site is conserved among Schlafen family members, their enzymatic activities differ (78).

SLFN11 was first shown to inhibit human immunodeficiency virus type 1 (HIV-1) translation in a codon-discrimination manner, preferentially targeting RNAs with atypical A/U-biased codon usage. This restriction requires tRNA binding, although the precise molecular mechanism remains incompletely understood (81).

Flaviviral genomes display codon usage patterns that differ from those of highly expressed host genes, making their translation sensitive to perturbations in tRNA availability or ribosome–tRNA interactions (82–84). In cells expressing SLFN11, translation of flaviviral RNAs is impaired in a codon-dependent manner, resulting in reduced viral translation and decreased viral infectivity, while viral RNA levels remain largely unchanged (85). SLFN11 has been shown to inhibit WNV, ZIKV, and DENV in glioblastoma cell models, with restriction requiring the N-terminal portion containing the Schlafen core domain (85). This activity likely involves, at least in part, preventing virus-induced modifications of the host tRNA repertoire that could otherwise enhance translation and folding of the viral polyprotein. Although SLFN11 is the most studied family member in this context, other Schlafens may also modulate flavivirus replication (78). For example, SLFN13 cleaves the acceptor stem of tRNAs and can moderately restrict ZIKV replication (86) (Fig. 3B).

SLFN11 demonstrates how the IFN response exploits subtle differences in codon usage and tRNA availability to selectively inhibit viral translation. Its activity highlights the importance of translation elongation as a point of vulnerability for RNA viruses such as flaviviruses. Identifying how SLFN11 alters tRNA availability and codon translation, and defining the basis for viral selectivity, will provide key insight into codon-dependent antiviral control and may inform strategies to exploit this vulnerability therapeutically.

### SAMD9L

SAMD9 and its paralog SAMD9L are ISGs with broad antiviral activity and essential roles in cellular homeostasis. They arose from an ancient gene duplication event and share a similar domain architecture, with approximately 60% amino-acid sequence identity (87, 88). In humans, both proteins are involved in the control of protein synthesis and cell proliferation, and gain-of-function mutations in SAMD9 or SAMD9L cause severe genetic disorders linked to excessive translation inhibition, underscoring their central role in regulating cellular translation (89, 90).

SAMD9 and SAMD9L have emerged as important antiviral effectors against a diverse range of viruses. Their antiviral activity is well-established in the context of poxvirus infection, where both human SAMD9 and SAMD9L, as well as murine SAMD9L, restrict viral replication (91–93). In addition, human SAMD9L restricts lentiviruses (94), whereas inhibition of reoviruses and rotaviruses has been reported for human SAMD9 and murine SAMD9L (95). Consistent with these observations, both proteins harbor a Schlafen-like ribonuclease domain (94), and recent work has identified SAMD9 as an anticodon nuclease (ACNase), specifically cleaving tRNA^Phe^, leading to global translation inhibition (93). Although the precise target of SAMD9L is unknown, it is likely to cleave specific tRNAs in a similar manner, contributing to its activity as a translation inhibitor. These findings place SAMD9 and SAMD9L within a broader group of IFN-stimulated antiviral factors, including SLFN11, that restrict viral replication by targeting the host translation machinery.

Our group has identified SAMD9L as a restriction factor for flaviviruses. SAMD9L overexpression inhibits replication of WNV, ZIKV, DENV, and USUV, whereas SAMD9L knockdown partially relieves IFN-mediated restriction of WNV in myeloid cells (96). In contrast, SAMD9 does not restrict flavivirus replication in the context of infection. Mechanistically, SAMD9L inhibits viral protein synthesis without altering viral RNA levels, indicating that it restricts flavivirus infection at the level of translation rather than affecting RNA stability or replication. This restriction requires the Schlafen-like ribonuclease domain, as loss-of-function mutations in this domain abolish antiviral activity (96) (Fig. 3B).

As observed for other translation-targeting ISGs, the antiviral activity of SAMD9L is strongly influenced by the RNA context. Viral RNAs expressed within a flaviviral replicon are markedly more sensitive to SAMD9L than reporter RNAs expressed from plasmids, suggesting that features intrinsic to flaviviral RNAs condition SAMD9L responsiveness (96). These may include highly structured UTRs, conserved internal RNA elements, or long-range RNA interactions characteristic of flaviviral genomes, although the precise determinants remain to be identified.

Interestingly, although SAMD9 does not restrict flavivirus translation during viral replication, it can repress the translation of flaviviral RNAs when they are directly transfected into cells (96). This indicates that SAMD9 has the intrinsic capacity to inhibit flaviviral RNA translation outside the context of infection. This observation suggests that the distinct specificities of SAMD9 and SAMD9L arise from differences in their activation or regulation during infection rather than from differences in RNA targets. In line with this idea, SAMD9 activity has been shown to be triggered during poxvirus infection (93), raising the possibility that SAMD9 remains inactive during flavivirus replication or that flaviviruses selectively counteract SAMD9 but not SAMD9L (96).

Finally, in addition to their direct antiviral activity, SAMD9 and SAMD9L can also function as innate immune sensors (95). Both proteins have been shown to detect cytosolic double-stranded nucleic acids and initiate innate immune signaling, acting as cytoplasmic pattern recognition receptors (95). As for IFIT and Schlafen proteins, this dual role highlights the integration of RNA sensing and translational control within the IFN response. However, in the context of flavivirus infection, our data indicate that SAMD9L restricts viral replication primarily through direct inhibition of viral RNA translation, independently of IFN signaling or secondary ISG induction (96).

Despite progress in characterizing SAMD9L as a flavivirus restriction factor, the mechanism underlying its selective inhibition of viral translation remains largely unknown. Key questions include which tRNAs or other targets are affected, how SAMD9L is specifically activated during flavivirus infection, and why SAMD9 does not exhibit the same antiviral effect. Elucidating these molecular details will be critical to understanding flavivirus-specific inhibition of translation and the broader role of Schlafen-like proteins in antiviral defense.

## INTERFERON-STIMULATED GENES THAT INDIRECTLY BLOCK TRANSLATION BY REDUCING VIRAL RNA ABUNDANCE

Several ISGs do not inhibit translation directly but instead act upstream by sharply reducing the amount of viral RNA available for protein synthesis. In these cases, the dominant effect on translation is secondary to RNA degradation or destabilization. Nevertheless, the outcome is a potent suppression of viral translation and a strong restriction of replication.

### The OAS–RNase L pathway

The oligoadenylate synthetase (OAS)–RNase L pathway is a classical IFN-induced antiviral mechanism that suppresses viral replication by degrading RNA and reducing translation. OAS proteins are activated by dsRNA, a common viral replication intermediate, and catalyze the synthesis of 2′–5′ oligoadenylates (2–5A) from ATP (97). These small molecules induce dimerization and activation of RNase L, a ubiquitous endoribonuclease that cleaves single-stranded RNA (ssRNA), rapidly degrading RNA and suppressing protein synthesis (97).

In mice, the OAS family comprises eight Oas1 genes (Oas1a–h), Oas2, Oas3, and two Oas-like genes (Oasl1 and Oasl2) (98). Oas1b is a major determinant of flavivirus resistance. Mice carrying truncating mutations are highly susceptible to WNV and YFV (99), whereas expression of full-length Oas1b partially protects neurons and fibroblasts by limiting early viral RNA accumulation (100, 101). This protective effect appears largely independent of RNase L, although RNase L itself can restrict WNV in mouse embryonic fibroblasts and *in vivo* through OAS-independent mechanisms (34, 102).

In humans, the OAS family includes OAS1, OAS2, OAS3, and the OAS-like protein OASL, all strongly induced by type I IFNs. Alternative splicing generates multiple isoforms with distinct C-terminal sequences, subcellular localization, and functional properties (98, 103). OAS1 produces five isoforms (p42, p44, p46, p48, and p52), OAS2 two isoforms (p69 and p71), and OAS3 a single isoform (p100) (98). These differences influence their capacity to activate RNase L and mediate antiviral responses.

**Fig 4:**
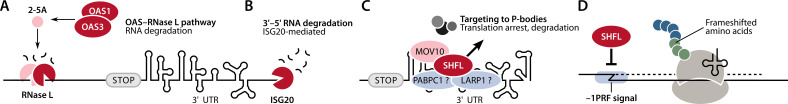
ISGs limiting *Orthoflavivirus* RNA abundance. (**A**) Activation of OAS1 and OAS3 by viral double-stranded RNA induces synthesis of 2′–5′ oligoadenylates (2–5A), which activate RNase L. RNase L cleaves viral RNA, reducing genome abundance and generating RNA fragments that stimulate innate immune signaling. Antiviral activity varies among orthoflaviviruses. (**B**) The 3′–5′ exonuclease ISG20 degrades viral genomic RNA and replication intermediates. Structural elements within the 3′ UTR can block ISG20-mediated degradation. (**C**) SHFL binds flavivirus genomic RNA at the 3′ UTR and interacts with host RNA-binding proteins, including PABPC1, LARP1, and the RNA helicase MOV10. Through these associations, SHFL can influence viral RNA stability, modulate recruitment of translation factors, and is associated with relocalization of viral RNA into RNA granules such as P-bodies or related RNP structures, contributing to reduced viral protein synthesis. Question marks indicate that direct binding of PABPC1 and LARP1 to flaviviral RNA within this complex has not been formally demonstrated. (**D**) SHFL suppresses the −1 frameshift used by certain orthoflaviviruses to produce proteins from overlapping coding sequences.

OAS3 is the main enzyme responsible for synthesizing 2–5A and activating RNase L in response to dsRNA, and is both necessary and sufficient for this pathway (104, 105). The OAS3/RNase L pathway exhibits antiviral activity against flaviviruses, including DENV-2 and WNV (106, 107) (Fig. 4A). Human OAS1 isoforms p42 and p46 also contribute to restriction of DENV-2 (106), and a SNP shifting splicing from p46 to p52 correlates with higher WNV titers, highlighting the role of specific isoforms in early viral control (108) (Fig. 4A). In addition, both murine and human OAS1 can exert RNase L-independent antiviral functions (109). OAS1 binds AU-rich elements in specific host mRNAs, including IFNβ mRNA, thereby enhancing their stability and translation despite global translational shutdown. This activity contributes to antiviral defense, including against WNV (109).

Mechanistic studies in WNV-infected cells show that viral replication generates dsRNA that accumulates in ER-associated replication organelles. Here, OAS3 condenses on dsRNA to form cytoplasmic foci known as double-stranded RNA-induced foci (dRIF), which create high local dsRNA concentrations and facilitate rapid RNase L activation (110). These condensates act as molecular switches in antiviral signaling and provide a structural explanation for WNV’s sensitivity to the OAS3/RNase L pathway (34, 102, 110). In contrast, its antiviral activity is markedly weaker against other flaviviruses (111, 112). During DENV infection, viral RNAs largely escape RNase L-mediated decay, allowing viral protein synthesis to continue despite host mRNA degradation (111). In ZIKV infection, RNase L activity does not reduce infectious virus production and can even support replication factory assembly through its non-enzymatic functions (107). In all cases, RNase L preferentially targets host mRNAs while largely sparing viral RNAs, including those of DENV-2, ZIKV, and WNV, indicating that its antiviral effects are largely mediated through host mRNA depletion and translational inhibition, rather than direct targeting of viral RNA (113).

Despite considerable advances, key questions remain regarding the OAS–RNase L pathway, including viral sensitivity, RNase L selectivity, and the contributions of individual OAS isoforms. How the spatial organization of OAS3 and RNase L influences antiviral efficiency and how flaviviruses evade this pathway remains to be clarified. Addressing these issues could inform strategies to enhance OAS–RNase L-mediated antiviral defenses.

### ISG20

Since its discovery in 1997 in Daudi cells as a novel ISG (114), ISG20 has emerged as a potent broad-spectrum antiviral effector. It has been shown to inhibit a diverse array of viruses, including, but not limited to, members of the *Retroviridae*, *Orthomyxoviridae*, *Rhabdoviridae*, *Picornaviridae*, *Hepadnaviridae*, *Togaviridae*, and *Flaviviridae* (32, 70, 115–123). Functionally, ISG20 is a 3′–5′ RNA exonuclease of the DEDDh superfamily that degrades nucleic acids with a strong preference for single-stranded RNA (124). This enzymatic specificity explains why ISG20 efficiently targets so many unrelated RNA viruses as well as viruses whose life cycle involves RNA intermediates (Fig. 4B).

The direct degradation of viral RNA by ISG20 has been demonstrated for several viruses, including HIV-1 and hepatitis B virus (HBV) (117, 125). Unsurprisingly, some viruses have evolved strategies to evade ISG20-mediated inhibition. For instance, HIV-1 RNA incorporates internal 2′-O-methylations via the host methyltransferase FTSJ3, which protects the genome from degradation (117). Beyond its well-established role in RNA degradation, emerging evidence indicates that ISG20 can also inhibit translation of viral RNAs in the absence of RNA degradation, although this activity still depends on its RNase activity (120).

In the context of flavivirus infection, ISG20 has been shown to restrict replication of viruses such as DENV and WNV, primarily by reducing viral RNA levels, thereby indirectly limiting translation (32, 121, 122). In a recent study, our group demonstrated that this sensitivity results from the direct degradation of their viral RNA genomes by ISG20 (70). In the same work, USUV was identified as a notable exception, as it is resistant to ISG20-mediated restriction. We further showed that ISG20 is unable to degrade the USUV genome due to a specific secondary structure within the DB2 element in the viral 3′ UTR, which shields the genome from exonucleolytic attack. This particular structure is both necessary and sufficient to confer resistance to ISG20, as its insertion into WNV, normally susceptible, makes it refractory to ISG20 (70).

Altogether, these findings highlight the broad-spectrum antiviral activity of ISG20, which restricts the replication of most flaviviruses through viral genome degradation, although some viral RNAs may also be subject to translational inhibition (126). Highly stable stem-loop structures, such as the one found at the 3′ end of Mopeia virus RNA, a member of the *Arenaviridae* family, can impede ISG20-mediated degradation (127). This makes the susceptibility of flavivirus genomes, which also feature a highly conserved stem-loop at the 3′ extremity, particularly intriguing, since it would be expected to confer protection against ISG20 (70). Recent studies have revealed that secondary structures, RNA epitranscriptomic modifications, and RNA-binding proteins all contribute to defining RNA sensitivity to ISG20 (126). It is likely that the fine balance between these elements, and possibly others, determines whether a given RNA is sensitive or resistant to ISG20 (126). Future research will be needed to fully understand the factors governing ISG20 susceptibility across viral RNAs.

### Shiftless

Shiftless (SHFL), encoded by the IFN-stimulated gene *C19orf66* and also referred to as IRAV or RyDEN, is a multifunctional antiviral factor with broad activity against RNA viruses, including flaviviruses. Initially identified as a restriction factor for DENV (128), SHFL was later shown to modulate programmed −1 ribosomal frameshifting (−1PRF), a translational recoding mechanism exploited by several viruses, including HIV-1 (129).

SHFL restricts replication of all four DENV serotypes as well as WNV, ZIKV, YFV, JEV, and USUV (70, 128, 130–133). *In vivo*, murine SHFL limits ZIKV replication and pathology, reducing viral loads and neuroinflammation, highlighting its physiological relevance during flavivirus infection (134).

Multiple, non-mutually exclusive mechanisms contribute to SHFL-mediated flavivirus restriction. SHFL associates with viral RNAs and components of viral replication complexes, as well as with host RNA-binding proteins, including PABPC1 and LARP1, leading to impaired viral translation and decreased viral RNA abundance. These observations suggest that SHFL affects viral RNA stability, ribosome access, or both (128, 130, 134) (Fig. 4C).

SHFL has also been reported to interact with the IFN-stimulated RNA helicase MOV10, a component of the RNA-induced silencing complex (RISC), which enhances SHFL antiviral activity against DENV (130). As suggested by Balinsky et al., SHFL can re-localize viral RNA to cytoplasmic processing bodies (P-bodies), sites of RNA decay, thereby promoting translation inhibition or degradation of viral transcripts (130) (Fig. 4C).

In addition to its effects on RNA metabolism, SHFL can directly interfere with viral translation by inhibiting −1PRF. This mechanism is best characterized for JEV, where a −1PRF event is required for synthesis of the extended NS1′ protein (135). SHFL inhibits this frameshifting event, leading to reduced NS1’ expression and impaired viral replication (133) (Fig. 4D).

Finally, SHFL has been shown to promote lysosome-dependent degradation of viral proteins, including NS3 during ZIKV and JEV infection, adding a post-translational layer to its antiviral activity (132, 133).

Collectively, these studies indicate that SHFL acts as a versatile antiviral effector whose mode of action varies depending on the flavivirus and cellular context. Rather than functioning through a single dominant mechanism, SHFL appears to restrict flavivirus replication by coordinating effects on viral RNA metabolism, ribosome behavior, and viral protein stability, ultimately converging on a strong reduction of viral protein expression.

Despite progress in understanding SHFL’s antiviral functions, the relative contributions of its multiple mechanisms and the determinants of virus- and host-specificity remain unclear. How SHFL is recruited to replication complexes, P-bodies, or ribosomes and how it interacts with other ISGs are important open questions. Investigating these aspects and exploring strategies to enhance SHFL activity could reveal new host-directed antiviral approaches.

## CONCLUSION AND PERSPECTIVES

Flaviviruses depend entirely on the host translation machinery to express their single polyprotein from a capped, structured RNA genome that must simultaneously support replication, translation, and genome cyclization. These overlapping functional constraints limit the evolutionary flexibility of viral RNA elements and restrict the range of escape options available to counter translation-targeting ISGs, likely at a significant fitness cost.

As discussed throughout this review, host cells exploit this vulnerability through diverse ISGs that interfere with distinct but interconnected steps of the translation process, including initiation factor recruitment, ribosome loading, elongation dynamics, polysome association, and viral RNA stability. Rather than inducing a global shutdown of protein synthesis, many of these effectors selectively target viral RNAs, positioning translation as a central interface of host–flavivirus conflict. The impact of individual ISGs varies substantially between flaviviruses, reflecting differences in RNA structures, sequence composition, and viral countermeasures, and highlighting the tight interplay between viral RNA features and host defense mechanisms.

A defining feature of ISG-mediated inhibition of translation is its specificity. Several ISGs discriminate viral from cellular RNAs by sensing cap structures, nucleotide composition, codon usage, RNA modifications, or higher-order RNA structures, enabling potent antiviral activity while preserving host translation. However, the molecular basis of this selectivity remains incompletely understood. Dissecting how ISGs distinguish viral transcripts from host mRNAs, and how this discrimination operates across cellular contexts, represents a major challenge for the field and a prerequisite to fully understand selective antiviral control.

In response to this selective pressure, flaviviruses have evolved strategies to mitigate translational inhibition. Viral proteins can modulate stress response pathways, remodel RNA–protein interactions, or exploit RNA modifications and structural elements to maintain ribosome engagement under restrictive conditions. In addition, some flaviviruses sustain translation when cap-dependent initiation is compromised, through non-canonical mechanisms involving long-range RNA interactions and host factors (20, 38–41), although their contribution to resistance against ISG-mediated restriction remains unclear. These countermeasures underscore that translation is a dynamic interface shaped by reciprocal evolutionary pressures, where escape from one restriction mechanism may incur costs at other essential steps of the viral life cycle.

Despite this progress, important questions remain regarding the coordination and specificity of translation-targeting ISGs. How do multiple translation-targeting ISGs act together? Do they cooperate, compete, or interfere? How does cell type–specific expression shape viral tropism and disease outcome? Addressing these questions will require integrated approaches combining virology, RNA biology, and quantitative analyses of translation in infected cells.

In this context, while individual ISGs targeting flavivirus translation have been extensively characterized, their combinatorial activity remains poorly understood. Available data suggest a layered organization in which broadly expressed ISGs, such as PKR, RNase L, ZAP, and IFIT1, mediate rapid early restriction across multiple cell types (136–140), whereas factors such as SLFN11, SAMD9L, SHFL, and ISG20 display more context-dependent activity, reinforcing or fine-tuning antiviral responses, particularly in immune cells (96, 141–143). This organization supports a model in which both cell identity and temporal regulation shape antiviral outcomes and determine virus-specific sensitivity to the IFN response.

Recent advances in high-resolution and quantitative approaches now offer powerful opportunities to dissect these mechanisms in greater detail. Ribosome profiling (Ribo-seq) provides genome-wide snapshots of ribosome occupancy, distinguishing effects on initiation, elongation, or ribosome stalling. Applied to infected cells, it can reveal how individual ISGs or combinations thereof reshape ribosome distribution on viral versus host RNAs. For example, recent work on ZIKV demonstrated that upstream open reading frames in the 5′ UTR may modulate ribosome engagement on the main ORF, partially counteracting eIF2α-mediated translational repression (144), while other Ribo-seq studies showed that PKR activation selectively alters ribosome occupancy on viral RNAs (36). Complementary approaches, including polysome profiling (145, 146), quantitative proteomics (113), and single-molecule translation imaging (147, 148), will further refine our understanding of flavivirus translation dynamics in real time. Together, these methods provide a framework to address many of the remaining questions about how translation-targeting ISGs function during infection.

Finally, while direct therapeutic use of ISGs is complicated by their pleiotropic roles, the vulnerabilities they exploit provide a roadmap for selective antiviral strategies. Targeting virus-specific dependencies in translation, such as non-canonical initiation mechanisms, codon or tRNA constraints, or evasion of RNA surveillance, could yield interventions that are both potent and selective. Integrating mechanistic insights from classical molecular studies with modern quantitative approaches will be key to developing strategies that leverage translation-targeting vulnerabilities without broadly impairing host protein synthesis.

Taken together, these findings establish translation not merely as a step in the flavivirus life cycle, but as a central and multifaceted battleground where host defenses exploit intrinsic constraints of viral RNA to achieve selective and robust antiviral control. We propose that these constraints define a limited adaptive landscape in which escape from one ISG may increase sensitivity to others, thereby shaping the evolution of flaviviruses under IFN pressure.

## References

[1] Pierson TC, Diamond MS. 2020. The continued threat of emerging flaviviruses. Nat Microbiol 5:796–812. doi:10.1038/s41564-020-0714-0 32367055 PMC7696730

[2] Cadar D, Simonin Y. 2022. Human usutu virus infections in Europe: a new risk on horizon? Viruses 15:77. doi:10.3390/v15010077 36680117 PMC9866956

[3] Hamel R, Phanitchat T, Wichit S, Morales Vargas RE, Jaroenpool J, Diagne CT, Pompon J, Missé D. 2021. New insights into the biology of the emerging tembusu virus. Pathogens 10:1010. doi:10.3390/pathogens10081010 34451474 PMC8398659

[4] White SK, Lednicky JA, Okech BA, Morris JG, Dunford JC. 2018. Spondweni virus in field-caught culex quinquefasciatus mosquitoes, Haiti, 2016. Emerg Infect Dis 24:1765–1767. doi:10.3201/eid2409.171957 30124422 PMC6106418

[5] Ng WC, Soto-Acosta R, Bradrick SS, Garcia-Blanco MA, Ooi EE. 2017. The 5’ and 3’ untranslated regions of the flaviviral genome. Viruses 9:137. doi:10.3390/v9060137 28587300 PMC5490814

[6] Chapman EG, Costantino DA, Rabe JL, Moon SL, Wilusz J, Nix JC, Kieft JS. 2014. The structural basis of pathogenic subgenomic flavivirus RNA (sfRNA) production. Science 344:307–310. doi:10.1126/science.1250897 24744377 PMC4163914

[7] Manokaran G, Finol E, Wang C, Gunaratne J, Bahl J, Ong EZ, Tan HC, Sessions OM, Ward AM, Gubler DJ, Harris E, Garcia-Blanco MA, Ooi EE. 2015. Dengue subgenomic RNA binds TRIM25 to inhibit interferon expression for epidemiological fitness. Science 350:217–221. doi:10.1126/science.aab3369 26138103 PMC4824004

[8] Mukhopadhyay S, Kuhn RJ, Rossmann MG. 2005. A structural perspective of the flavivirus life cycle. Nat Rev Microbiol 3:13–22. doi:10.1038/nrmicro1067 15608696

[9] Schneider WM, Chevillotte MD, Rice CM. 2014. Interferon-stimulated genes: a complex web of host defenses. Annu Rev Immunol 32:513–545. doi:10.1146/annurev-immunol-032713-120231 24555472 PMC4313732

[10] Schoggins JW. 2019. Interferon-stimulated genes: what do they all do? Annu Rev Virol 6:567–584. doi:10.1146/annurev-virology-092818-015756 31283436

[11] Hinnebusch AG, Lorsch JR. 2012. The mechanism of eukaryotic translation initiation: new insights and challenges. Cold Spring Harb Perspect Biol 4:4. doi:10.1101/cshperspect.a011544 PMC3475172 22815232

[12] Martineau Y, Derry MC, Wang X, Yanagiya A, Berlanga JJ, Shyu A-B, Imataka H, Gehring K, Sonenberg N. 2008. Poly(A)-binding protein-interacting protein 1 binds to eukaryotic translation initiation factor 3 to stimulate translation. Mol Cell Biol 28:6658–6667. doi:10.1128/MCB.00738-08 18725400 PMC2573229

[13] Ramanathan A, Robb GB, Chan S-H. 2016. mRNA capping: biological functions and applications. Nucleic Acids Res 44:7511–7526. doi:10.1093/nar/gkw551 27317694 PMC5027499

[14] Eiermann N, Haneke K, Sun Z, Stoecklin G, Ruggieri A. 2020. Dance with the devil: stress granules and signaling in antiviral responses. Viruses 12:984. doi:10.3390/v12090984 32899736 PMC7552005

[15] Williams TD, Rousseau A. 2024. Translation regulation in response to stress. FEBS J 291:5102–5122. doi:10.1111/febs.17076 38308808 PMC11616006

[16] Egloff M-P, Benarroch D, Selisko B, Romette J-L, Canard B. 2002. An RNA cap (nucleoside-2′-O-)-methyltransferase in the flavivirus RNA polymerase NS5: crystal structure and functional characterization. EMBO J 21:2757–2768. doi:10.1093/emboj/21.11.2757 12032088 PMC125380

[17] Issur M, Geiss BJ, Bougie I, Picard-Jean F, Despins S, Mayette J, Hobdey SE, Bisaillon M. 2009. The flavivirus NS5 protein is a true RNA guanylyltransferase that catalyzes a two-step reaction to form the RNA cap structure. RNA 15:2340–2350. doi:10.1261/rna.1609709 19850911 PMC2779676

[18] Ray D, Shah A, Tilgner M, Guo Y, Zhao Y, Dong H, Deas TS, Zhou Y, Li H, Shi P-Y. 2006. West Nile virus 5′-cap structure is formed by sequential guanine N-7 and ribose 2′-O methylations by nonstructural protein 5. J Virol 80:8362–8370. doi:10.1128/JVI.00814-06 16912287 PMC1563844

[19] Fernández-Sanlés A, Ríos-Marco P, Romero-López C, Berzal-Herranz A. 2017. Functional information stored in the conserved structural RNA domains of flavivirus genomes. Front Microbiol 8:546. doi:10.3389/fmicb.2017.00546 28421048 PMC5376627

[20] Berzal-Herranz A, Berzal-Herranz B, Ramos-Lorente SE, Romero-López C. 2022. The Genomic 3′ UTR of Flaviviruses Is a Translation Initiation Enhancer. Int J Mol Sci 23:8604. doi:10.3390/ijms23158604 35955738 PMC9369090

[21] Polacek C, Friebe P, Harris E. 2009. Poly(A)-binding protein binds to the non-polyadenylated 3′ untranslated region of dengue virus and modulates translation efficiency. J Gen Virol 90:687–692. doi:10.1099/vir.0.007021-0 19218215

[22] Galabru J, Hovanessian AG. 1985. Two interferon-induced proteins are involved in the protein kinase complex dependent on double-stranded RNA. Cell 43:685–694. doi:10.1016/0092-8674(85)90241-7 2416468

[23] Meurs E, Chong K, Galabru J, Thomas NS, Kerr IM, Williams BR, Hovanessian AG. 1990. Molecular cloning and characterization of the human double-stranded RNA-activated protein kinase induced by interferon. Cell 62:379–390. doi:10.1016/0092-8674(90)90374-n 1695551

[24] Krishnamoorthy T, Pavitt GD, Zhang F, Dever TE, Hinnebusch AG. 2001. Tight binding of the phosphorylated alpha subunit of initiation factor 2 (eIF2alpha) to the regulatory subunits of guanine nucleotide exchange factor eIF2B is required for inhibition of translation initiation. Mol Cell Biol 21:5018–5030. doi:10.1128/MCB.21.15.5018-5030.2001 11438658 PMC87228

[25] Sudhakar A, Ramachandran A, Ghosh S, Hasnain SE, Kaufman RJ, Ramaiah KV. 2000. Phosphorylation of serine 51 in initiation factor 2 alpha (eIF2 alpha) promotes complex formation between eIF2 alpha(P) and eIF2B and causes inhibition in the guanine nucleotide exchange activity of eIF2B. Biochemistry 39:12929–12938. doi:10.1021/bi0008682 11041858

[26] Pakos-Zebrucka K, Koryga I, Mnich K, Ljujic M, Samali A, Gorman AM. 2016. The integrated stress response. EMBO Rep 17:1374–1395. doi:10.15252/embr.201642195 27629041 PMC5048378

[27] García MA, Meurs EF, Esteban M. 2007. The dsRNA protein kinase PKR: virus and cell control. Biochimie 89:799–811. doi:10.1016/j.biochi.2007.03.001 17451862

[28] Peña J, Harris E. 2011. Dengue virus modulates the unfolded protein response in a time-dependent manner. J Biol Chem 286:14226–14236. doi:10.1074/jbc.M111.222703 21385877 PMC3077624

[29] Basu M, Courtney SC, Brinton MA. 2017. Arsenite-induced stress granule formation is inhibited by elevated levels of reduced glutathione in West Nile virus-infected cells. PLoS Pathog 13:e1006240. doi:10.1371/journal.ppat.1006240 28241074 PMC5344523

[30] Blázquez A-B, Martín-Acebes MA, Poderoso T, Saiz J-C. 2021. Relevance of oxidative stress in inhibition of eIF2 alpha phosphorylation and stress granules formation during Usutu virus infection. PLoS Negl Trop Dis 15:e0009072. doi:10.1371/journal.pntd.0009072 33493202 PMC7861526

[31] Tu Y-C, Yu C-Y, Liang J-J, Lin E, Liao C-L, Lin Y-L. 2012. Blocking double-stranded RNA-activated protein kinase PKR by Japanese encephalitis virus nonstructural protein 2A. J Virol 86:10347–10358. doi:10.1128/JVI.00525-12 22787234 PMC3457255

[32] Jiang D, Weidner JM, Qing M, Pan X-B, Guo H, Xu C, Zhang X, Birk A, Chang J, Shi P-Y, Block TM, Guo J-T. 2010. Identification of five interferon-induced cellular proteins that inhibit west nile virus and dengue virus infections. J Virol 84:8332–8341. doi:10.1128/JVI.02199-09 20534863 PMC2916517

[33] Li J, Ding SC, Cho H, Chung BC, Gale M, Chanda SK, Diamond MS. 2013. A short hairpin RNA screen of interferon-stimulated genes identifies a novel negative regulator of the cellular antiviral response. mBio 4:e00385–00313. doi:10.1128/mBio.00385-13 23781071 PMC3684836

[34] Samuel MA, Whitby K, Keller BC, Marri A, Barchet W, Williams BRG, Silverman RH, Gale M, Diamond MS. 2006. PKR and RNase L contribute to protection against lethal west nile virus infection by controlling early viral spread in the periphery and replication in neurons. J Virol 80:7009–7019. doi:10.1128/JVI.00489-06 16809306 PMC1489062

[35] Elbahesh H, Scherbik SV, Brinton MA. 2011. West Nile virus infection does not induce PKR activation in rodent cells. Virology (Auckl) 421:51–60. doi:10.1016/j.virol.2011.08.008 PMC3208726 21982595

[36] Pallarés HM, González López Ledesma MM, Oviedo-Rouco S, Castellano LA, Costa Navarro GS, Fernández-Alvarez AJ, D’Andreiz MJ, Aldas-Bulos VD, Alvarez DE, Bazzini AA, Gamarnik AV. 2024. Zika virus non-coding RNAs antagonize antiviral responses by PKR-mediated translational arrest. Nucleic Acids Res 52:11128–11147. doi:10.1093/nar/gkae507 38917323 PMC11472168

[37] Ricciardi-Jorge T, da Rocha EL, Gonzalez-Kozlova E, Rodrigues-Luiz GF, Ferguson BJ, Sweeney T, Irigoyen N, Mansur DS. 2023. PKR-mediated stress response enhances dengue and Zika virus replication. mBio 14:e0093423. doi:10.1128/mbio.00934-23 37732809 PMC10653888

[38] Wang T, Merits A, Wu Y, Wang M, Jia R, Zhu D, Liu M, Zhao X, Yang Q, Wu Y, Zhang S, Liu Y, Zhang L, Yu Y, Pan L, Chen S, Cheng A. 2020. cis-acting sequences and secondary structures in untranslated regions of duck tembusu virus RNA are important for cap-independent translation and viral proliferation. J Virol 94:e00906-20. doi:10.1128/JVI.00906-20 32522848 PMC7394898

[39] Song Y, Mugavero J, Stauft CB, Wimmer E. 2019. Dengue and zika virus 5′ untranslated regions harbor internal ribosomal entry site functions. mBio 10:e00459–19. doi:10.1128/mBio.00459-19 30967466 PMC6456755

[40] Li C, Zhang L, Tang C, Chen X, Shi J, Li Q, Jiao X, Guo J, Wang B, Bu K, Wahaab A, Yuan Y, Sun M-A, Li Y. 2025. DDX3 regulates the cap‐independent translation of the japanese encephalitis virus via its interactions with PABP1 and the untranslated regions of the viral genome. Adv Sci (Weinh) 12:e2502493. doi:10.1002/advs.202502493 40344524 PMC12279179

[41] Edgil D, Polacek C, Harris E. 2006. Dengue virus utilizes a novel strategy for translation initiation when cap-dependent translation is inhibited. J Virol 80:2976–2986. doi:10.1128/JVI.80.6.2976-2986.2006 16501107 PMC1395423

[42] Diamond MS, Farzan M. 2013. The broad-spectrum antiviral functions of IFIT and IFITM proteins. Nat Rev Immunol 13:46–57. doi:10.1038/nri3344 23237964 PMC3773942

[43] Levy D, Larner A, Chaudhuri A, Babiss LE, Darnell JE. 1986. Interferon-stimulated transcription: isolation of an inducible gene and identification of its regulatory region. Proc Natl Acad Sci USA 83:8929–8933. doi:10.1073/pnas.83.23.8929 3466167 PMC387047

[44] Wathelet M, Moutschen S, Defilippi P, Cravador A, Collet M, Huez G, Content J. 1986. Molecular cloning, full-length sequence and preliminary characterization of a 56-kDa protein induced by human interferons. Eur J Biochem 155:11–17. doi:10.1111/j.1432-1033.1986.tb09452.x 3753936

[45] Daffis S, Szretter KJ, Schriewer J, Li J, Youn S, Errett J, Lin T-Y, Schneller S, Zust R, Dong H, Thiel V, Sen GC, Fensterl V, Klimstra WB, Pierson TC, Buller RM, Gale M, Shi P-Y, Diamond MS. 2010. 2′-O methylation of the viral mRNA cap evades host restriction by IFIT family members. Nature 468:452–456. doi:10.1038/nature09489 21085181 PMC3058805

[46] Habjan M, Hubel P, Lacerda L, Benda C, Holze C, Eberl CH, Mann A, Kindler E, Gil-Cruz C, Ziebuhr J, Thiel V, Pichlmair A. 2013. Sequestration by IFIT1 impairs translation of 2′O-unmethylated capped RNA. PLoS Pathog 9:e1003663. doi:10.1371/journal.ppat.1003663 24098121 PMC3789756

[47] Kimura T, Katoh H, Kayama H, Saiga H, Okuyama M, Okamoto T, Umemoto E, Matsuura Y, Yamamoto M, Takeda K. 2013. Ifit1 inhibits Japanese encephalitis virus replication through binding to 5′ capped 2′-O unmethylated RNA. J Virol 87:9997–10003. doi:10.1128/JVI.00883-13 23824812 PMC3754022

[48] Kumar P, Sweeney TR, Skabkin MA, Skabkina OV, Hellen CUT, Pestova TV. 2014. Inhibition of translation by IFIT family members is determined by their ability to interact selectively with the 5′-terminal regions of cap0-, cap1- and 5′ppp- mRNAs. Nucleic Acids Res 42:3228–3245. doi:10.1093/nar/gkt1321 24371270 PMC3950709

[49] Johnson B, VanBlargan LA, Xu W, White JP, Shan C, Shi P-Y, Zhang R, Adhikari J, Gross ML, Leung DW, Diamond MS, Amarasinghe GK. 2018. Human IFIT3 modulates IFIT1 RNA binding specificity and protein stability. Immunity 48:487–499. doi:10.1016/j.immuni.2018.01.014 29525521 PMC6251713

[50] Szretter KJ, Daniels BP, Cho H, Gainey MD, Yokoyama WM, Gale M, Virgin HW, Klein RS, Sen GC, Diamond MS. 2012. 2′-O Methylation of the viral mRNA cap by west nile virus evades Ifit1-dependent and -independent mechanisms of host restriction in vivo. PLoS Pathog 8:e1002698. doi:10.1371/journal.ppat.1002698 22589727 PMC3349756

[51] Li S-H, Dong H, Li X-F, Xie X, Zhao H, Deng Y-Q, Wang X-Y, Ye Q, Zhu S-Y, Wang H-J, Zhang B, Leng Q-B, Zuest R, Qin E-D, Qin C-F, Shi P-Y. 2013. Rational design of a flavivirus vaccine by abolishing viral RNA 2′-O methylation. J Virol 87:5812–5819. doi:10.1128/JVI.02806-12 23487465 PMC3648161

[52] Züst R, Dong H, Li X-F, Chang DC, Zhang B, Balakrishnan T, Toh Y-X, Jiang T, Li S-H, Deng Y-Q, Ellis BR, Ellis EM, Poidinger M, Zolezzi F, Qin C-F, Shi P-Y, Fink K. 2013. Rational design of a live attenuated dengue vaccine: 2′-o-methyltransferase mutants are highly attenuated and immunogenic in mice and macaques. PLoS Pathog 9:e1003521. doi:10.1371/journal.ppat.1003521 23935499 PMC3731252

[53] Guo J, Hui DJ, Merrick WC, Sen GC. 2000. A new pathway of translational regulation mediated by eukaryotic initiation factor 3. EMBO J 19:6891–6899. doi:10.1093/emboj/19.24.6891 11118224 PMC305884

[54] Wang C, Pflugheber J, Sumpter R, Sodora DL, Hui D, Sen GC, Gale M. 2003. Alpha interferon induces distinct translational control programs to suppress hepatitis C virus RNA replication. J Virol 77:3898–3912. doi:10.1128/jvi.77.7.3898-3912.2003 12634350 PMC150642

[55] Yang Z, Liang H, Zhou Q, Li Y, Chen H, Ye W, Chen D, Fleming J, Shu H, Liu Y. 2012. Crystal structure of ISG54 reveals a novel RNA binding structure and potential functional mechanisms. Cell Res 22:1328–1338. doi:10.1038/cr.2012.111 22825553 PMC3434343

[56] Cho H, Shrestha B, Sen GC, Diamond MS. 2013. A role for Ifit2 in restricting West Nile virus infection in the brain. J Virol 87:8363–8371. doi:10.1128/JVI.01097-13 23740986 PMC3719802

[57] Hsu Y-L, Shi S-F, Wu W-L, Ho L-J, Lai J-H. 2013. Protective roles of interferon-induced protein with tetratricopeptide repeats 3 (IFIT3) in dengue virus infection of human lung epithelial cells. PLoS One 8:e79518. doi:10.1371/journal.pone.0079518 24223959 PMC3817122

[58] Wichit S, Hamel R, Zanzoni A, Diop F, Cribier A, Talignani L, Diack A, Ferraris P, Liegeois F, Urbach S, Ekchariyawat P, Merits A, Yssel H, Benkirane M, Missé D. 2019. SAMHD1 enhances chikungunya and zika virus replication in human skin fibroblasts. IJMS 20:1695. doi:10.3390/ijms20071695 30959732 PMC6480247

[59] Ficarelli M, Neil SJD, Swanson CM. 2021 a. Targeted restriction of viral gene expression and replication by the ZAP antiviral system. Annu Rev Virol 8:265–283. doi:10.1146/annurev-virology-091919-104213 34129371

[60] Ficarelli M, Neil SJD, Swanson CM. 2021. Targeted restriction of viral gene expression and replication by the ZAP antiviral system. Annu Rev Virol 8:265–283. doi:10.1146/annurev-virology-091919-104213 34129371

[61] Shao R, Visser I, Fros JJ, Yin X. 2024. Versatility of the zinc-finger antiviral protein (ZAP) as a modulator of viral infections. Int J Biol Sci 20:4585–4600. doi:10.7150/ijbs.98029 39309436 PMC11414379

[62] Gao G, Guo X, Goff SP. 2002. Inhibition of retroviral RNA production by ZAP, a CCCH-type zinc finger protein. Science 297:1703–1706. doi:10.1126/science.1074276 12215647

[63] Chiu H-P, Chiu H, Yang C-F, Lee Y-L, Chiu F-L, Kuo H-C, Lin R-J, Lin Y-L. 2018. Inhibition of Japanese encephalitis virus infection by the host zinc-finger antiviral protein. PLoS Pathog 14:e1007166. doi:10.1371/journal.ppat.1007166 30016363 PMC6049953

[64] Gonzalez-Perez AC, Stempel M, Wyler E, Urban C, Piras A, Hennig T, Ganskih S, Wei Y, Heim A, Landthaler M, Pichlmair A, Dölken L, Munschauer M, Erhard F, Brinkmann MM. 2021. The zinc finger antiviral protein ZAP restricts human cytomegalovirus and selectively binds and destabilizes viral UL4/UL5 transcripts. mBio 12:e02683-20. doi:10.1128/mBio.02683-20 33947766 PMC8263000

[65] Meagher JL, Takata M, Gonçalves-Carneiro D, Keane SC, Rebendenne A, Ong H, Orr VK, MacDonald MR, Stuckey JA, Bieniasz PD, Smith JL. 2019. Structure of the zinc-finger antiviral protein in complex with RNA reveals a mechanism for selective targeting of CG-rich viral sequences. Proc Natl Acad Sci USA 116:24303–24309. doi:10.1073/pnas.1913232116 31719195 PMC6883784

[66] Takata MA, Gonçalves-Carneiro D, Zang TM, Soll SJ, York A, Blanco-Melo D, Bieniasz PD. 2017. CG dinucleotide suppression enables antiviral defence targeting non-self RNA. Nature 550:124–127. doi:10.1038/nature24039 28953888 PMC6592701

[67] Guo X, Ma J, Sun J, Gao G. 2007. The zinc-finger antiviral protein recruits the RNA processing exosome to degrade the target mRNA. Proc Natl Acad Sci USA 104:151–156. doi:10.1073/pnas.0607063104 17185417 PMC1765426

[68] Zhu Y, Wang X, Goff SP, Gao G. 2012. Translational repression precedes and is required for ZAP-mediated mRNA decay. EMBO J 31:4236–4246. doi:10.1038/emboj.2012.271 23023399 PMC3492732

[69] Zhu Y, Chen G, Lv F, Wang X, Ji X, Xu Y, Sun J, Wu L, Zheng Y-T, Gao G. 2011. Zinc-finger antiviral protein inhibits HIV-1 infection by selectively targeting multiply spliced viral mRNAs for degradation. Proc Natl Acad Sci USA 108:15834–15839. doi:10.1073/pnas.1101676108 21876179 PMC3179061

[70] Zoladek J, El Kazzi P, Caval V, Vivet-Boudou V, Cannac M, Davies EL, Rossi S, Bribes I, Rouilly L, Simonin Y, Jouvenet N, Decroly E, Paillart J-C, Wilson SJ, Nisole S. 2024. A specific domain within the 3’ untranslated region of Usutu virus confers resistance to the exonuclease ISG20. Nat Commun 15:8528. doi:10.1038/s41467-024-52870-w 39358425 PMC11447015

[71] Le NPK, Singh PP, Sabir AJ, Trus I, Karniychuk U. 2024. Endogenous ZAP is associated with altered Zika virus infection phenotype. Virol J 21:285. doi:10.1186/s12985-024-02557-x 39522048 PMC11549788

[72] Sabir AJ, Le NPK, Singh PP, Karniychuk U. 2024. Endogenous ZAP affects Zika virus RNA interactome. RNA Biol 21:1–10. doi:10.1080/15476286.2024.2388911 PMC11352719 39183472

[73] Yang E, Nguyen LP, Wisherop CA, Kan RL, Li MMH. 2022. The role of ZAP and TRIM25 RNA binding in restricting viral translation. Front Cell Infect Microbiol 12:886929. doi:10.3389/fcimb.2022.886929 35800389 PMC9253567

[74] Michalski D, Ontiveros JG, Russo J, Charley PA, Anderson JR, Heck AM, Geiss BJ, Wilusz J. 2019. Zika virus noncoding sfRNAs sequester multiple host-derived RNA-binding proteins and modulate mRNA decay and splicing during infection. Journal of Biological Chemistry 294:16282–16296. doi:10.1074/jbc.RA119.009129 31519749 PMC6827284

[75] Moon SL, Anderson JR, Kumagai Y, Wilusz CJ, Akira S, Khromykh AA, Wilusz J. 2012. A noncoding RNA produced by arthropod-borne flaviviruses inhibits the cellular exoribonuclease XRN1 and alters host mRNA stability. RNA 18:2029–2040. doi:10.1261/rna.034330.112 23006624 PMC3479393

[76] Metzner FJ, Wenzl SJ, Kugler M, Krebs S, Hopfner K-P, Lammens K. 2022. Mechanistic understanding of human SLFN11. Nat Commun 13:5464. doi:10.1038/s41467-022-33123-0 36115853 PMC9482658

[77] Li M, Kao E, Malone D, Gao X, Wang JYJ, David M. 2018. DNA damage-induced cell death relies on SLFN11-dependent cleavage of distinct type II tRNAs. Nat Struct Mol Biol 25:1047–1058. doi:10.1038/s41594-018-0142-5 30374083 PMC6579113

[78] Kim ET, Weitzman MD. 2022. Schlafens can put viruses to sleep. Viruses 14:442. doi:10.3390/v14020442 35216035 PMC8875196

[79] Jo U, Pommier Y. 2022. Structural, molecular, and functional insights into Schlafen proteins. Exp Mol Med 54:730–738. doi:10.1038/s12276-022-00794-0 35768579 PMC9256597

[80] Al-Marsoummi S, Vomhof-DeKrey EE, Basson MD. 2021. Schlafens: emerging proteins in cancer cell biology. Cells 10:2238. doi:10.3390/cells10092238 34571887 PMC8465726

[81] Li M, Kao E, Gao X, Sandig H, Limmer K, Pavon-Eternod M, Jones TE, Landry S, Pan T, Weitzman MD, David M. 2012. Codon-usage-based inhibition of HIV protein synthesis by human schlafen 11. Nature 491:125–128. doi:10.1038/nature11433 23000900 PMC3705913

[82] Castellano LA, McNamara RJ, Pallarés HM, Gamarnik AV, Alvarez DE, Bazzini AA. 2024. Dengue virus preferentially uses human and mosquito non-optimal codons. Mol Syst Biol 20:1085–1108. doi:10.1038/s44320-024-00052-7 39039212 PMC11450187

[83] Chin W-X, Kong HY, Zhu IXY, Teo ZY, Faruk R, Lee RCH, Ho SX, Aw ZQ, Yi B, Hou XJ, Tan AKY, Yogarajah T, Huber RG, Cai Y, Wan Y, Chu JJH. 2023. Flavivirus genome recoding by codon optimisation confers genetically stable in vivo attenuation in both mice and mosquitoes. PLoS Pathog 19:e1011753. doi:10.1371/journal.ppat.1011753 37883598 PMC10629665

[84] Wang H, Liu S, Zhang B, Wei W. 2016. Analysis of synonymous codon usage bias of zika virus and its adaption to the hosts. PLoS One 11:e0166260. doi:10.1371/journal.pone.0166260 27893824 PMC5125587

[85] Valdez F, Salvador J, Palermo PM, Mohl JE, Hanley KA, Watts D, Llano M. 2019. Schlafen 11 restricts flavivirus replication. J Virol 93:e00104–19. doi:10.1128/JVI.00104-19 31118262 PMC6639263

[86] Yang J-Y, Deng X-Y, Li Y-S, Ma X-C, Feng J-X, Yu B, Chen Y, Luo Y-L, Wang X, Chen M-L, Fang Z-X, Zheng F-X, Li Y-P, Zhong Q, Kang T-B, Song L-B, Xu R-H, Zeng M-S, Chen W, Zhang H, Xie W, Gao S. 2018. Structure of Schlafen13 reveals a new class of tRNA/rRNA- targeting RNase engaged in translational control. Nat Commun 9:1165. doi:10.1038/s41467-018-03544-x 29563550 PMC5862951

[87] Legrand A, Demeure R, Chantharath A, Rey C, Baltenneck J, Gilchrist CLM, Rocha JL, Loyer C, Picard L, Cimarelli A, Steinegger M, Rousset F, Sudmant PH, Etienne L. 2025. Evolutionary characterization of antiviral SAMD9/9L across kingdoms supports ancient convergence and lineage-specific adaptations. Nat Ecol Evol 9:2206–2222. doi:10.1038/s41559-025-02845-x 41219459 PMC12680541

[88] Lemos de Matos A, Liu J, McFadden G, Esteves PJ. 2013. Evolution and divergence of the mammalian SAMD9/SAMD9L gene family. BMC Evol Biol 13:121. doi:10.1186/1471-2148-13-121 23758988 PMC3685527

[89] Mekhedov SL, Makarova KS, Koonin EV. 2017. The complex domain architecture of SAMD9 family proteins, predicted STAND-like NTPases, suggests new links to inflammation and apoptosis. Biol Direct 12:13. doi:10.1186/s13062-017-0185-2 28545555 PMC5445408

[90] Peng S, Meng X, Zhang F, Pathak PK, Chaturvedi J, Coronado J, Morales M, Mao Y, Qian S-B, Deng J, Xiang Y. 2022. Structure and function of an effector domain in antiviral factors and tumor suppressors SAMD9 and SAMD9L. Proc Natl Acad Sci USA 119:e2116550119. doi:10.1073/pnas.2116550119 35046037 PMC8795524

[91] Liu J, McFadden G. 2015. SAMD9 is an innate antiviral host factor with stress response properties that can be antagonized by poxviruses. J Virol 89:1925–1931. doi:10.1128/JVI.02262-14 25428864 PMC4300762

[92] Meng X, Zhang F, Yan B, Si C, Honda H, Nagamachi A, Sun L-Z, Xiang Y. 2018. A paralogous pair of mammalian host restriction factors form a critical host barrier against poxvirus infection. PLoS Pathog 14:e1006884. doi:10.1371/journal.ppat.1006884 29447249 PMC5831749

[93] Zhang F, Ji Q, Chaturvedi J, Morales M, Mao Y, Meng X, Dong L, Deng J, Qian S-B, Xiang Y. 2023. Human SAMD9 is a poxvirus-activatable anticodon nuclease inhibiting codon-specific protein synthesis. Sci Adv 9. doi:10.1126/sciadv.adh8502 PMC10246899 37285440

[94] Legrand A, Dahoui C, De La Myre Mory C, Noy K, Guiguettaz L, Versapuech M, Loyer C, Pillon M, Wcislo M, Guéguen L, Berlioz-Torrent C, Cimarelli A, Mateo M, Fiorini F, Ricci EP, Etienne L. 2024. SAMD9L acts as an antiviral factor against HIV-1 and primate lentiviruses by restricting viral and cellular translation. PLoS Biol 22:e3002696. doi:10.1371/journal.pbio.3002696 38959200 PMC11221667

[95] Hou G, Beatty W, Ren L, Ooi YS, Son J, Zhu Y, Sheng Q, Huang W, Li D, Liu C, Welsh OL, Sutherland DM, Dermody TS, Shen C, Liu J, Sibley LD, Ding S. 2025. SAMD9 senses cytosolic double-stranded nucleic acids in epithelial and mesenchymal cells to induce antiviral immunity. Nat Commun 16:3756. doi:10.1038/s41467-025-59090-w 40263291 PMC12015307

[96] Cannac M, Zoladek J, Bribes I, Fresneau--Resende M, Legrand A, Demeure R, Zusinaite E, Merits A, Etienne L, Nisole S. 2025. SAMD9L inhibits flavivirus translation independently of its capacity to trigger innate immune response. PLoS Pathog 21:e1013773. doi:10.1371/journal.ppat.1013773 41359666 PMC12698002

[97] Silverman RH. 2007. Viral encounters with 2′,5′-oligoadenylate synthetase and rnase l during the interferon antiviral response. J Virol 81:12720–12729. doi:10.1128/JVI.01471-07 17804500 PMC2169107

[98] Sarkar SN, Harioudh MK, Shao L, Perez J, Ghosh A. 2023. The many faces of oligoadenylate synthetases. J Interferon Cytokine Res 43:487–494. doi:10.1089/jir.2023.0098 37751211 PMC10654648

[99] Mashimo T, Lucas M, Simon-Chazottes D, Frenkiel M-P, Montagutelli X, Ceccaldi P-E, Deubel V, Guenet J-L, Despres P. 2002. A nonsense mutation in the gene encoding 2′-5′-oligoadenylate synthetase/L1 isoform is associated with West Nile virus susceptibility in laboratory mice. Proc Natl Acad Sci USA 99:11311–11316. doi:10.1073/pnas.172195399 12186974 PMC123253

[100] Kajaste-Rudnitski A, Mashimo T, Frenkiel M-P, Guénet J-L, Lucas M, Desprès P. 2006. The 2′,5′-oligoadenylate synthetase 1b is a potent inhibitor of west nile virus replication inside infected cells. Journal of Biological Chemistry 281:4624–4637. doi:10.1074/jbc.M508649200 16371364

[101] Lucas M, Mashimo T, Frenkiel M, Simon‐Chazottes D, Montagutelli X, Ceccaldi P, Guénet J, Desprès P. 2003. Infection of mouse neurones by West Nile virus is modulated by the interferon‐inducible 2′‐5′ oligoadenylate synthetase 1b protein. Immunol Cell Biol 81:230–236. doi:10.1046/j.1440-1711.2003.01166.x 12752688

[102] Scherbik SV, Paranjape JM, Stockman BM, Silverman RH, Brinton MA. 2006. RNase L plays a role in the antiviral response to west nile virus. J Virol 80:2987–2999. doi:10.1128/JVI.80.6.2987-2999.2006 16501108 PMC1395436

[103] Di H, Elbahesh H, Brinton MA. 2020. Characteristics of human OAS1 isoform proteins. Viruses 12:152. doi:10.3390/v12020152 32013110 PMC7077331

[104] Li Y, Banerjee S, Wang Y, Goldstein SA, Dong B, Gaughan C, Silverman RH, Weiss SR. 2016. Activation of RNase L is dependent on OAS3 expression during infection with diverse human viruses. Proc Natl Acad Sci USA 113:2241–2246. doi:10.1073/pnas.1519657113 26858407 PMC4776461

[105] Ibsen MS, Gad HH, Thavachelvam K, Boesen T, Desprès P, Hartmann R. 2014. The 2′-5′-oligoadenylate synthetase 3 enzyme potently synthesizes the 2′-5′-oligoadenylates required for RNase L activation. J Virol 88:14222–14231. doi:10.1128/JVI.01763-14 25275129 PMC4249133

[106] Lin R-J, Yu H-P, Chang B-L, Tang W-C, Liao C-L, Lin Y-L. 2009. Distinct antiviral roles for human 2′,5′-oligoadenylate synthetase family members against dengue virus infection. J Immunol 183:8035–8043. doi:10.4049/jimmunol.0902728 19923450

[107] Whelan JN, Parenti NA, Hatterschide J, Renner DM, Li Y, Reyes HM, Dong B, Perez ER, Silverman RH, Weiss SR. 2021. Zika virus employs the host antiviral RNase L protein to support replication factory assembly. Proc Natl Acad Sci USA 118:e2101713118. doi:10.1073/pnas.2101713118 34031250 PMC8179202

[108] Lim JK, Lisco A, McDermott DH, Huynh L, Ward JM, Johnson B, Johnson H, Pape J, Foster GA, Krysztof D, Follmann D, Stramer SL, Margolis LB, Murphy PM. 2009. Genetic variation in OAS1 is a risk factor for initial infection with west nile virus in man. PLoS Pathog 5:e1000321. doi:10.1371/journal.ppat.1000321 19247438 PMC2642680

[109] Harioudh MK, Perez J, Chong Z, Nair S, So L, McCormick KD, Ghosh A, Shao L, Srivastava R, Soveg F, Ebert TS, Atianand MK, Hornung V, Savan R, Diamond MS, Sarkar SN. 2024. Oligoadenylate synthetase 1 displays dual antiviral mechanisms in driving translational shutdown and protecting interferon production. Immunity 57:446–461. doi:10.1016/j.immuni.2024.02.002 38423012 PMC10939734

[110] Cusic R, Burke JM. 2024. Condensation of RNase L promotes its rapid activation in response to viral infection in mammalian cells. Sci Signal 17:eadi9844. doi:10.1126/scisignal.adi9844 38771918 PMC11391522

[111] Burke JM, Gilchrist AR, Sawyer SL, Parker R. 2021. RNase L limits host and viral protein synthesis via inhibition of mRNA export. Sci Adv 7. doi:10.1126/sciadv.abh2479 PMC8177694 34088676

[112] Madden JC, Cui D, Brinton MA. 2019. RNase L antiviral activity is not a critical component of the Oas1b-mediated flavivirus resistance phenotype. J Virol 93:e00946–19. doi:10.1128/JVI.00946-19 PMC6819929 31462564

[113] Watkins JM, Douglas CJ, Cusic R, Seath CP, Burke JM. 2026. RNase L regulates the antiviral proteome by accelerating mRNA decay, inhibiting nuclear mRNA export, and repressing transcription. Cell Rep 45:116924. doi:10.1016/j.celrep.2026.116924 41758647 PMC13206644

[114] Gongora C, David G, Pintard L, Tissot C, Hua TD, Dejean A, Mechti N. 1997. Molecular cloning of a new interferon-induced PML nuclear body-associated protein. J Biol Chem 272:19457–19463. doi:10.1074/jbc.272.31.19457 9235947

[115] Weiss CM, Trobaugh DW, Sun C, Lucas TM, Diamond MS, Ryman KD, Klimstra WB. 2018. The interferon-induced exonuclease ISG20 exerts antiviral activity through upregulation of type I interferon response proteins. mSphere 3:e00209-18. doi:10.1128/mSphere.00209-18 30232164 PMC6147134

[116] Espert L, Degols G, Lin Y-L, Vincent T, Benkirane M, Mechti N. 2005. Interferon-induced exonuclease ISG20 exhibits an antiviral activity against human immunodeficiency virus type 1. J Gen Virol 86:2221–2229. doi:10.1099/vir.0.81074-0 16033969

[117] El Kazzi P, Rabah N, Chamontin C, Poulain L, Ferron F, Debart F, Canard B, Missé D, Coutard B, Nisole S, Decroly E. 2023. Internal RNA 2’O-methylation in the HIV-1 genome counteracts ISG20 nuclease-mediated antiviral effect. Nucleic Acids Res 51:2501–2515. doi:10.1093/nar/gkac996 36354007 PMC10085690

[118] Espert L, Degols G, Gongora C, Blondel D, Williams BR, Silverman RH, Mechti N. 2003. ISG20, a new interferon-induced RNase specific for single-stranded RNA, defines an alternative antiviral pathway against RNA genomic viruses. J Biol Chem 278:16151–16158. doi:10.1074/jbc.M209628200 12594219

[119] Qu H, Li J, Yang L, Sun L, Liu W, He H. 2016. Influenza A Virus-induced expression of ISG20 inhibits viral replication by interacting with nucleoprotein. Virus Genes 52:759–767. doi:10.1007/s11262-016-1366-2 27342813

[120] Wu N, Nguyen X-N, Wang L, Appourchaux R, Zhang C, Panthu B, Gruffat H, Journo C, Alais S, Qin J, Zhang N, Tartour K, Catez F, Mahieux R, Ohlmann T, Liu M, Du B, Cimarelli A. 2019. The interferon stimulated gene 20 protein (ISG20) is an innate defense antiviral factor that discriminates self versus non-self translation. PLoS Pathog 15:e1008093. doi:10.1371/journal.ppat.1008093 31600344 PMC6805002

[121] Zhou Z, Wang N, Woodson SE, Dong Q, Wang J, Liang Y, Rijnbrand R, Wei L, Nichols JE, Guo J-T, Holbrook MR, Lemon SM, Li K. 2011. Antiviral activities of ISG20 in positive-strand RNA virus infections. Virology (Auckl) 409:175–188. doi:10.1016/j.virol.2010.10.008 PMC3018280 21036379

[122] Ding J, Aldo P, Roberts CM, Stabach P, Liu H, You Y, Qiu X, Jeong J, Maxwell A, Lindenbach B, Braddock D, Liao A, Mor G. 2021. Placenta‐derived interferon‐stimulated gene 20 controls ZIKA virus infection. EMBO Reports 22. doi:10.15252/embr.202152450 PMC8490983 34405956

[123] Liu Y, Nie H, Mao R, Mitra B, Cai D, Yan R, Guo J-T, Block TM, Mechti N, Guo H. 2017. Interferon-inducible ribonuclease ISG20 inhibits hepatitis B virus replication through directly binding to the epsilon stem-loop structure of viral RNA. PLoS Pathog 13:e1006296. doi:10.1371/journal.ppat.1006296 28399146 PMC5388505

[124] Nguyen LH, Espert L, Mechti N, Wilson DM. 2001. The human interferon- and estrogen-regulated ISG20/HEM45 gene product degrades single-stranded RNA and DNA in vitro. Biochemistry 40:7174–7179. doi:10.1021/bi010141t 11401564

[125] Leong CR, Funami K, Oshiumi H, Mengao D, Takaki H, Matsumoto M, Aly HH, Watashi K, Chayama K, Seya T. 2016. Interferon-stimulated gene of 20 kDa protein (ISG20) degrades RNA of hepatitis B virus to impede the replication of HBV in vitro and in vivo. Oncotarget 7:68179–68193. doi:10.18632/oncotarget.11907 27626689 PMC5356548

[126] Zoladek J, Deymier S, Cimarelli A, Nisole S. 2025. Not all RNAs are created equal for the antiviral RNase ISG20. Trends Microbiol 33:583–585. doi:10.1016/j.tim.2025.01.014 39971662

[127] Louvat C, Deymier S, Nguyen X-N, Labaronne E, Noy K, Cariou M, Corbin A, Mateo M, Ricci EP, Fiorini F, Cimarelli A. 2024. Stable structures or PABP1 loading protects cellular and viral RNAs against ISG20-mediated decay. Life Sci Alliance 7:e202302233. doi:10.26508/lsa.202302233 38418089 PMC10902665

[128] Suzuki Y, Chin W-X, Han Q, Ichiyama K, Lee CH, Eyo ZW, Ebina H, Takahashi H, Takahashi C, Tan BH, Hishiki T, Ohba K, Matsuyama T, Koyanagi Y, Tan Y-J, Sawasaki T, Chu JJH, Vasudevan SG, Sano K, Yamamoto N. 2016. Characterization of RyDEN (C19orf66) as an interferon-stimulated cellular inhibitor against dengue virus replication. PLoS Pathog 12:e1005357. doi:10.1371/journal.ppat.1005357 26735137 PMC4703206

[129] Wang X, Xuan Y, Han Y, Ding X, Ye K, Yang F, Gao P, Goff SP, Gao G. 2019. Regulation of HIV-1 gag-pol expression by shiftless, an inhibitor of programmed -1 ribosomal frameshifting. Cell 176:625–635. doi:10.1016/j.cell.2018.12.030 30682371 PMC8486322

[130] Balinsky CA, Schmeisser H, Wells AI, Ganesan S, Jin T, Singh K, Zoon KC. 2017. IRAV (FLJ11286), an interferon-stimulated gene with antiviral activity against dengue virus, interacts with MOV10. J Virol 91:e01606-16. doi:10.1128/JVI.01606-16 27974568 PMC5309953

[131] Bribes I, Zoladek J, Cannac M, Salinas S, Wilson SJ, Nisole S. 2025. African strains of Zika virus resist ISG-mediated restriction. PLoS Negl Trop Dis 19:e0013326. doi:10.1371/journal.pntd.0013326 40658726 PMC12270304

[132] Wu Y, Yang X, Yao Z, Dong X, Zhang D, Hu Y, Zhang S, Lin J, Chen J, An S, Ye H, Zhang S, Qiu Z, He Z, Huang M, Wei G, Zhu X. 2020. C19orf66 interrupts Zika virus replication by inducing lysosomal degradation of viral NS3. PLoS Negl Trop Dis 14:e0008083. doi:10.1371/journal.pntd.0008083 32150556 PMC7082052

[133] Yu D, Zhao Y, Pan J, Yang X, Liang Z, Xie S, Cao R. 2021. C19orf66 inhibits japanese encephalitis virus replication by targeting -1 PRF and the NS3 Protein. Virol Sin 36:1443–1455. doi:10.1007/s12250-021-00423-6 34309824 PMC8692527

[134] Hanners NW, Mar KB, Boys IN, Eitson JL, De La Cruz-Rivera PC, Richardson RB, Fan W, Wight-Carter M, Schoggins JW. 2021. Shiftless inhibits flavivirus replication in vitro and is neuroprotective in a mouse model of Zika virus pathogenesis. Proc Natl Acad Sci USA 118:e2111266118. doi:10.1073/pnas.2111266118 34873063 PMC8670505

[135] Melian EB, Hinzman E, Nagasaki T, Firth AE, Wills NM, Nouwens AS, Blitvich BJ, Leung J, Funk A, Atkins JF, Hall R, Khromykh AA. 2010. NS1′ of flaviviruses in the japanese encephalitis virus serogroup is a product of ribosomal frameshifting and plays a role in viral neuroinvasiveness. J Virol 84:1641–1647. doi:10.1128/JVI.01979-09 19906906 PMC2812330

[136] Uhlén M, Fagerberg L, Hallström BM, Lindskog C, Oksvold P, Mardinoglu A, Sivertsson Å, Kampf C, Sjöstedt E, Asplund A, et al.. 2015. Tissue-based map of the human proteome. Science 347:1260419. doi:10.1126/science.1260419 25613900

[137] García MA, Gil J, Ventoso I, Guerra S, Domingo E, Rivas C, Esteban M. 2006. Impact of protein kinase PKR in cell biology: from antiviral to antiproliferative action. Microbiol Mol Biol Rev 70:1032–1060. doi:10.1128/MMBR.00027-06 17158706 PMC1698511

[138] Chakrabarti A, Jha BK, Silverman RH. 2011. New insights into the role of RNase L in innate immunity. J Interferon Cytokine Res 31:49–57. doi:10.1089/jir.2010.0120 21190483 PMC3021357

[139] Li MMH, Aguilar EG, Michailidis E, Pabon J, Park P, Wu X, de Jong YP, Schneider WM, Molina H, Rice CM, MacDonald MR. 2019. Characterization of novel splice variants of zinc finger antiviral protein (ZAP). J Virol 93:e00715-19. doi:10.1128/JVI.00715-19 31118263 PMC6714797

[140] McDermott JE, Vartanian KB, Mitchell H, Stevens SL, Sanfilippo A, Stenzel-Poore MP. 2012. Identification and validation of Ifit1 as an important innate immune bottleneck. PLoS One 7:e36465. doi:10.1371/journal.pone.0036465 22745654 PMC3380000

[141] Cheng J, Fu J, Tan Q, Liu Z, Guo K, Zhang L, He J, Zhou B, Liu X, Li D, Fu J. 2022. The regulation of ISG20 expression on SARS-CoV-2 infection in cancer patients and healthy individuals. Front Immunol 13:958898. doi:10.3389/fimmu.2022.958898 36177004 PMC9513371

[142] Puck A, Aigner R, Modak M, Cejka P, Blaas D, Stöckl J. 2015. Expression and regulation of Schlafen (SLFN) family members in primary human monocytes, monocyte-derived dendritic cells and T cells. Results Immunol 5:23–32. doi:10.1016/j.rinim.2015.10.001 26623250 PMC4625362

[143] Rodriguez W, Muller M. 2022. Shiftless, a critical piece of the innate immune response to viral infection. Viruses 14:1338. doi:10.3390/v14061338 35746809 PMC9230503

[144] Lefèvre C, Cook GM, Dinan AM, Torii S, Stewart H, Gibbons G, Nicholson AS, Echavarría-Consuegra L, Meredith LW, Lulla V, McGovern N, Kenyon JC, Goodfellow I, Deane JE, Graham SC, Lakatos A, Lambrechts L, Brierley I, Irigoyen N. 2024. Zika viruses encode 5′ upstream open reading frames affecting infection of human brain cells. Nat Commun 15:8822. doi:10.1038/s41467-024-53085-9 39394194 PMC11470053

[145] Roth H, Magg V, Uch F, Mutz P, Klein P, Haneke K, Lohmann V, Bartenschlager R, Fackler OT, Locker N, Stoecklin G, Ruggieri A. 2017. Flavivirus infection uncouples translation suppression from cellular stress responses. mBio 8:e02150-16. doi:10.1128/mBio.02150-16 28074025 PMC5225315

[146] Aviner R, Li KH, Frydman J, Andino R. 2021. Cotranslational prolyl hydroxylation is essential for flavivirus biogenesis. Nature 596:558–564. doi:10.1038/s41586-021-03851-2 34408324 PMC8789550

[147] Dave P, Roth G, Griesbach E, Mateju D, Hochstoeger T, Chao JA. 2023. Single-molecule imaging reveals translation-dependent destabilization of mRNAs. Mol Cell 83:589–606. doi:10.1016/j.molcel.2023.01.013 36731471 PMC9957601

[148] Mateju D, Eichenberger B, Voigt F, Eglinger J, Roth G, Chao JA. 2020. Single-molecule imaging reveals translation of mRNAs localized to stress granules. Cell 183:1801–1812. doi:10.1016/j.cell.2020.11.010 33308477

